# Effects of short-time exposure to atrazine on miRNA expression profiles in the gonad of common carp (*Cyprinus carpio*)

**DOI:** 10.1186/s12864-019-5896-6

**Published:** 2019-07-17

**Authors:** Fang Wang, Qian-wen Yang, Wen-Jie Zhao, Qi-Yan Du, Zhong-Jie Chang

**Affiliations:** College of Life Science, Henan Normal University, Xinxiang, Henan 453007 People’s Republic of China

**Keywords:** Atrazine, Targeting analysis, MicroRNA, Gonad development, *Cyprinus carpio*

## Abstract

**Background:**

Atrazine is widely used in agriculture and is a known endocrine disrupting chemical. Atrazine can seep into the water body through surface, posing a potential threat to the aquatic ecological environment and human drinking water source. In vertebrate, studies have shown that it can affect reproduction and development seriously, but its molecular mechanism for aquatic animals is unknown. Aquaculture is very common in China, especially common carp, whose females grow faster than males. However, the effects of atrazine on the reproduction of carp, especially miRNA, have not been investigated.

**Results:**

In this study, common carp (*Cyprinus carpio*) at two key developmental stages were exposed to atrazine in vitro. Sex ratio was observed to analyze the effect of atrazine on the sex. MiRNA expression profiles were analysed to identify miRNAs related to gonad development and to reveal the atrazine mechanisms interfering with gonad differentiation. The results showed that the sex ratio was biased towards females. Atrazine exposure caused significant alteration of multiple miRNAs. Predicted targets of differently-expressed miRNAs were involved in many reproductive biology signalling pathways.

**Conclusions:**

Our results indicate that atrazine promoted the expression of female-biased genes by decreasing miRNAs in primordial gonad. In addition, our results indicate that atrazine can up-regulate aromatase expression through miRNAs, which supports the hypothesis that atrazine has endocrine-disrupting activity by altering the gene expression profile of the Hypothalamus-Pituitary-Gonad axis through its corresponding miRNAs.

**Electronic supplementary material:**

The online version of this article (10.1186/s12864-019-5896-6) contains supplementary material, which is available to authorized users.

## Background

Sex determination in fish is significantly influenced by environmental factors, such as temperature, pH, exogenous hormones, and pollutants [1]. Pollutants, such as pesticides, are potential endocrine disruptors, which even at very low levels are sufficient to cause developmental and reproductive alterations in numerous species [2, 3].

With the development of agriculture, herbicides are increasingly used to avoid the manual removal of weeds, to reduce soil erosion, and to increase crop production rates [4]. However, the use of pesticides leads to serious harm to living organisms. Atrazine is a pre-emergent herbicide which is widely used on a variety of agricultural crops including sorghum grass, corn, wheat, and sugar cane, is frequently contaminating potable water supplies [5–8]. Atrazine alters male reproductive tissues when animals are exposed to during development, which makes it a suspected endocrine-disrupting chemical.

Various studies indicate that atrazine may act as a potential carcinogen, which adversely affects the neuroendocrine and reproductive systems [9–12]. Currently, the epigenetic, genetic, and cellular mechanisms altered by atrazine exposure are being studied [13–18]. Study in African clawed frogs (*Xenopus laevis*) showed that atrazine exposure, for as little as 48 h at 21 ppb, resulted in severe gonad dysgenesis [19]. Moreover, atrazine can induce hermaphroditism at concentrations of only 0.1 ppb [20]. In fish, exposure to atrazine can result in all-female sex ratio in zebrafish (*Danio rerio*) [21].

In zebrafish, atrazine exposure during embryonic development alters miRNAs, which play important roles in angiogenesis, cancer, and neurodevelopment [22]. Numerous studies have shown that atrazine has adverse effects on the neuroendocrine system, primarily affecting the hypothalamus–pituitary–gonad (HPG) axis. Atrazine decreases gonadotropin-releasing hormone release, the pre-ovulatory surge of luteinizing hormone, follicle stimulating hormone, and prolactin [17, 23–26]. However, the mechanism of action of atrazine is not well-understood.

MiRNAs is a class of single-stranded, highly conserved, non-coding RNA molecules of 19–24 nucleotides (nt), which regulate gene expression at the post-transcriptional level, by targeting specific sites in the 3′ untranslated region of mRNAs [27–29]. miRNAs play important roles in controlling multiple biological processes, such as cell proliferation and differentiation, embryonic development, apoptosis, cell cycle control, and immune and stress responses in various organs [30–34]. In the last few years, miRNAs have been reported to play an important role in the response to toxicant exposure and in the process of toxicant-induced tumorigenesis [35–37].

As a new tool for risk assessment, miRNAs can provide indications on the toxicology mechanisms associated with environmental factors and with disease. MiRNAs are also novel biomarkers of the diseases related to environmental factors [38]. Recently, an increasing number of studies have shown that miRNAs can functionally interact with a variety of environmental factors, such as drugs, viruses, radiation, and environmental chemicals [39–41]. Knowledge on the role miRNAs in toxicological responses is increasing, but is still limited.

As one of the most important cyprinid species, common carp, *Cyprinus carpio*, is widely cultivated [42]. Great progress has been made in the study of carp genomic recently. Common carp transcriptome was deep sequenced by Ji et al. (2012) and Jiang et al. (2016) [43, 44], who identified changes at the transcriptomic level in common carp spleen after 24 h of experimental infection with *Aeromonas hydrophila*. Using next generation sequencing, a large number of gene associated single-nucleotide polymorphisms (SNPs) as well as miRNA and miRNA-related SNPs were identified in four strains of common carp [45]. Studies also showed that miRNA-related SNPs can affect biogenesis and regulation in the common carp [46].

Yellow River carp is commercially important species in China, famous for its tender, tasty, and nutritional meat. Females grow faster than males, which makes the mechanism of sex differentiation and development an intriguing topic in this species [47, 48]. In our previous study, we profiled miRNAs from five different developmental stages of Yellow River carp, in order to identify differentially-expressed and novel miRNAs that may play important regulatory roles in ovary differentiation [49]. Our previous study showed that there is a dynamic shift in gene expression during gonad differentiation and development [50]. Environmental factors can affect miRNAs in fish, and even play a decisive role in some species.

Several studies have shown that in zebrafish and human atrazine exposure alters miRNAs associated with angiogenesis, cancer, and neurological development [51]. However, few studies have demonstrated the role of miRNAs in toxicological responses during sex differentiation and development in teleost fish.

In this study, we looked for correlations of miRNA and mRNA expressions during sex differentiation and development of carp, following atrazine exposure. The gonad development of carp has several critical periods, including primordial gonad and juvenile gonad. It would be valuable to understand the gene expression changes and the roles of miRNAs during the key stages of gonad development of carp, when they are exposed to atrazine. Therefore we aimed to investigate the effect of atrazine exposure on the global expression profile of miRNAs in the two key stages of gonad development by deep sequencing. We also predicted target genes that would affect gonad development. Our results would help us to better understand the molecular mechanisms of atrazine toxicity on gonad development, and to reveal the roles of miRNA–mRNA interactions in toxicological mechanisms, and the important impact on sex differentiation and gonad development of common carp.

## Results

### Effect of exposure to different concentration of atrazine on sex ratio

**Fig. 1:**
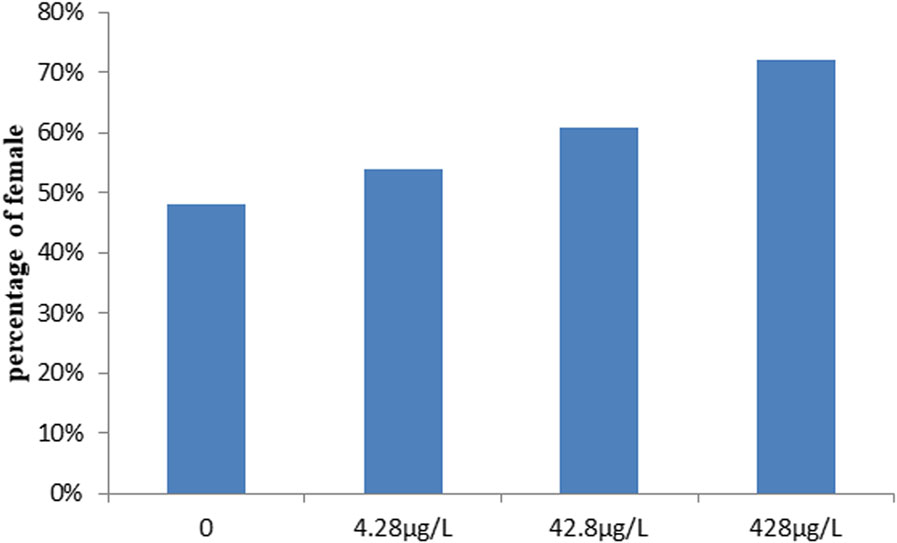
Percentage of female after exposed to different concentrations of atrazine

The gonads of carp were examined under a dissecting microscope to evaluate the sex at 130 dph. The results indicated that the effect of exposure to different concentration of atrazine on sex ratio in Yellow river carp were remarkable. The female percentage increased with increase of atrazine. When the concentration of atrazine in aquatic water was 0, 4.28, 42.8, or 428 μg/L, the female percentage were 48, 54, 61 and 72% respectively (Fig. 1).

### Construction of cDNA libraries for sequencing and small-RNA discovery

**Fig. 2:**
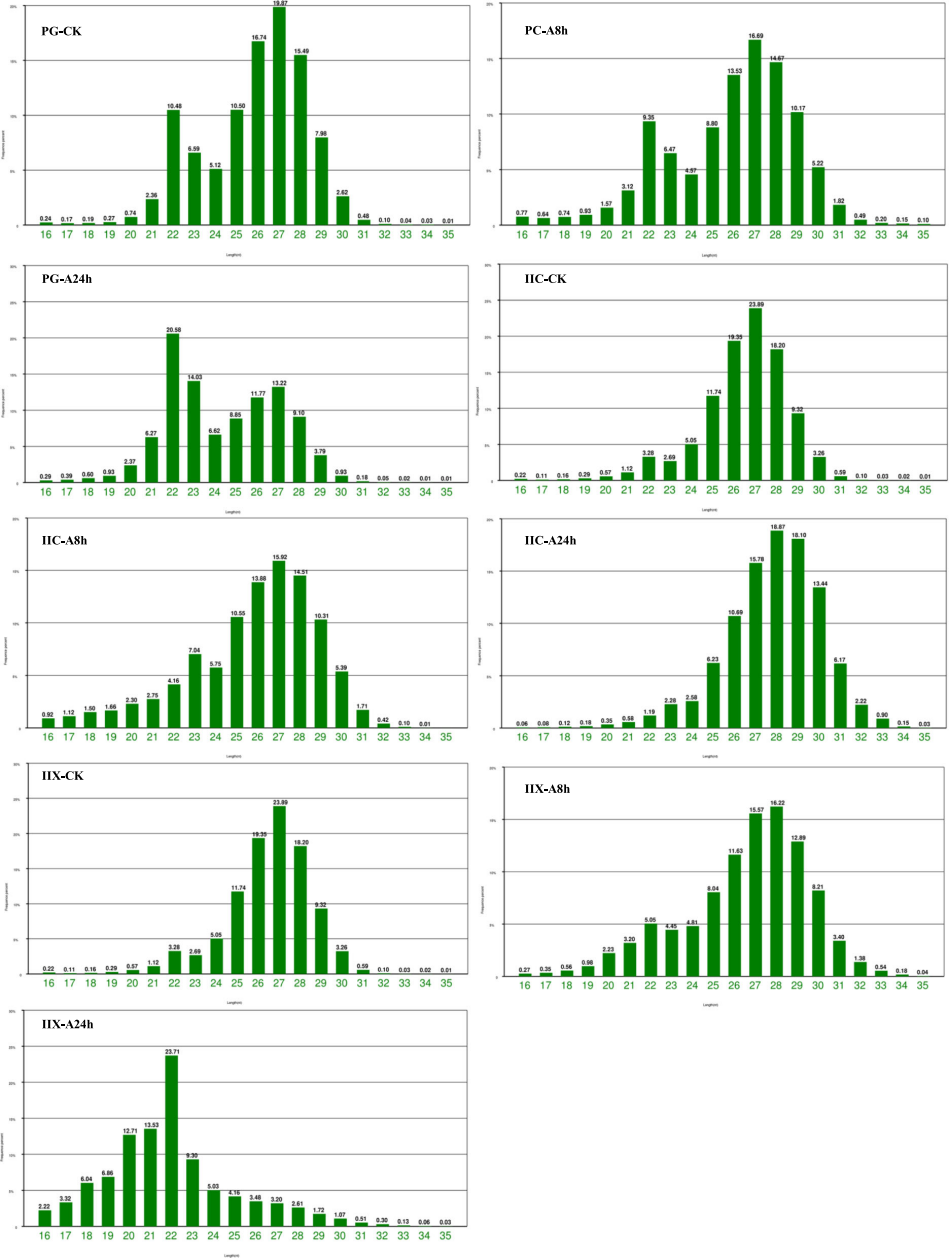
Length distribution of miRNA sequences from Yellow River carp in primordial gonad control (PG-CK), primordial gonad exposed to atrazine for 8 h (PG-A8h), primordial gonad exposed to atrazine for 24 h (PG-A24h), juvenile ovary control (IIC-CK), juvenile ovary exposed to atrazine for 8 h (IIC-A8h), juvenile ovary exposed to atrazine for 24 h (IIC-A24h), juvenile testis control (IIX-CK), juvenile testis exposed to atrazine for 8 h (IIX-A8h), juvenile testis exposed to atrazine for 24 h (IIX-A24h)

Nine cDNA libraries of small RNAs were constructed using pooled total RNAs from gonad tissues exposed to atrazine or control tissues collected from primordial gonad (PG) and from juvenile gonad stage carps. After filtering out low quality sequences, 5′ and 3′ adapters, and reads < 18 nt, A total of 10,281,292, 10,086,295, 11,985,647, 10,080,133, 11,724,632, 11,604,659, 11,502,749, 11,030,073, and 11,282,882 clean reads were obtained from the nine libraries. Solexa sequencing was then performed for further analysis (Table 1). we removed known types of RNA sequences including rRNA (3.84, 8.80, 8.84, 41.84, 16.31, 5.34, 0.85, 4.42 and 50.12%, respectively), tRNA (2.16%; 1.84%; 1.08%; 0.47%; 1.08%; 2.72%; 0.58%; 2.79%; and 0.37%), small nuclear RNA (snRNA), small nucleolar RNA (snoRNA), and repeat sequences after comparing the small-RNA sequences with NCBI GenBank and RFam. The clean reads of small RNAs from the nine libraries were mapped to the common carp genome with miRDeep2 software. A total of 4,895,831 (82.84%); 4,657,428 (84.16%); 7,083,013(84.91%); 5,536,128 (90.12%); 6,104,227 (86.69%); 5,627,337 (82.83%); 5,334,147 (79.12%); 5,337,365 (79.15%) and 7,463,759 (89.37%) of miRNA clean reads were mapped to the genome. The length distribution of the high-quality reads had different trends in the samples within the nine libraries. In the case of PG-CK samples, two peaks of length were observed at 22 nt and 27 nt. However, the size distribution of 21–23 nt increased and the size distribution of 26–29 nt decreased, after exposed to atrazine for 8 h and 24 h (Fig. 2). In the case of IIX-CK samples, higher miRNA mapped rates were observed in small RNAs of 26–28 nt in length. The size distribution of 21–23 nt increased and the size distribution of 26–29 nt decreased after exposure to atrazine for 8 h and 24 h (Fig. 2). In the case of IIC-CK samples, higher miRNA mapped rates were observed in small RNAs of 21–23 nt in length. The size distribution of 21–23 nt decreased and the size distribution of 27–29 nt increased after exposure to atrazine for 8 h and 24 h (Fig. 2). Small RNAs with length of 26–29 nt corresponded to Piwi-interacting RNAs (piRNAs) (Fig. 2), which are endogenous small non-coding RNA molecules 26–31 nt in length. Various studies have shown that Piwi–piRNA complexes have essential roles in gene silencing and in transposon regulation during germ cell differentiation and gonad development in animals [52–54].

**Table 1:**
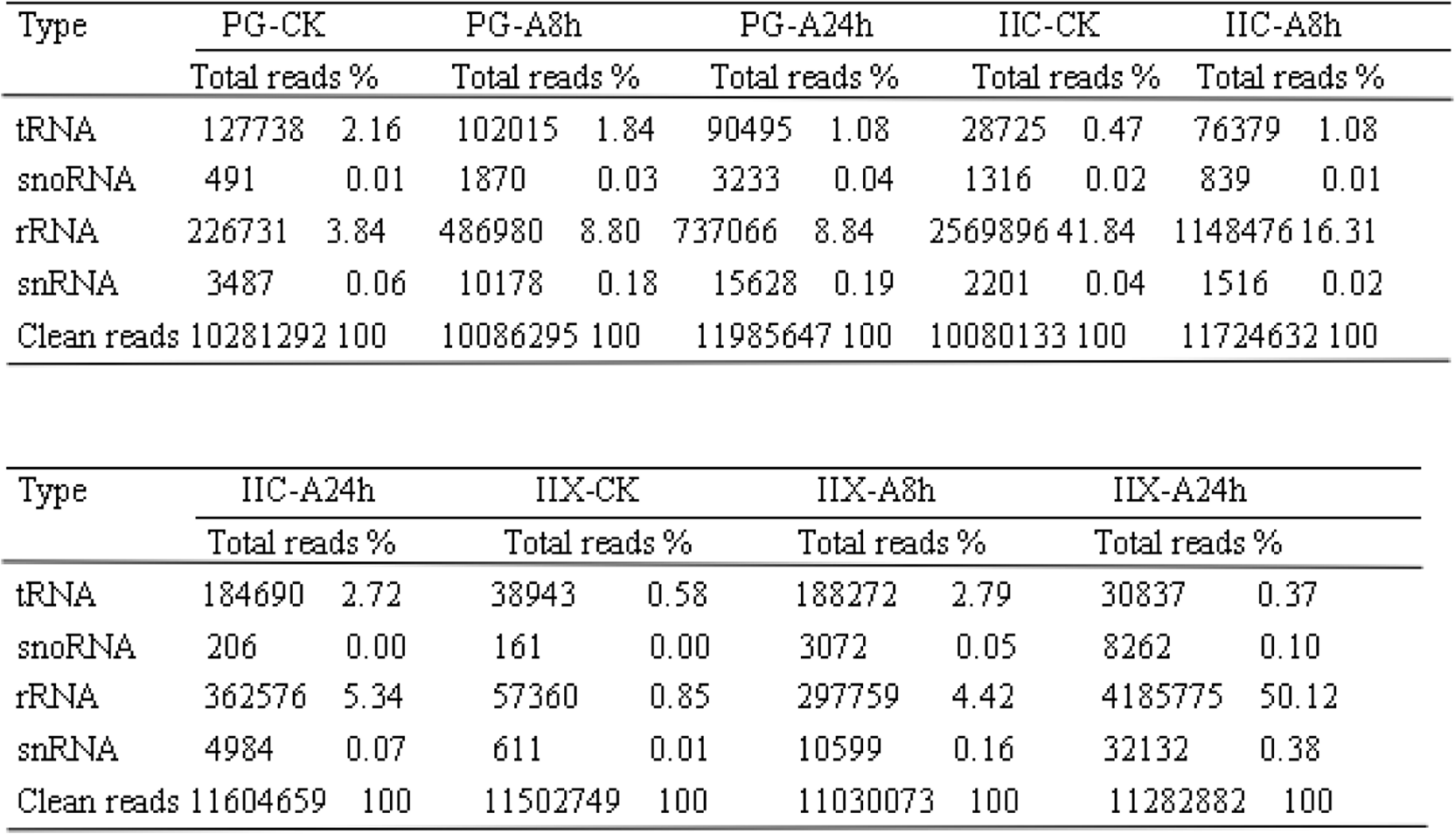
Distribution of sequenced clean reads

### Identification of miRNAs

To identify miRNAs in the gonad of the Yellow River carp exposed or not to atrazine, the clean reads were used and the miRNAs identified by comparison to the deposited miRNAs from miRBase. Mireap_v0.2 software was used for secondary structure prediction of novel miRNA. There was a total of 4443 miRNAs that were identified, including 3795 existing miRNAs, and 648 conserved miRNAs. Among the existing and conserved miRNAs, 7 miRNAs (ccr-miR-26a, ccr-miR-10b, ccr-miR-143, ccr-miR-181a, ccr-miR-100, ccr-miR-22a, and ccr-miR-92a) were the most abundant (TPM > 10,000) in all samples (TPM = Readout × 1,000,000 / Mapped reads).

### Validation of miRNAs with qPCR

**Fig. 3:**
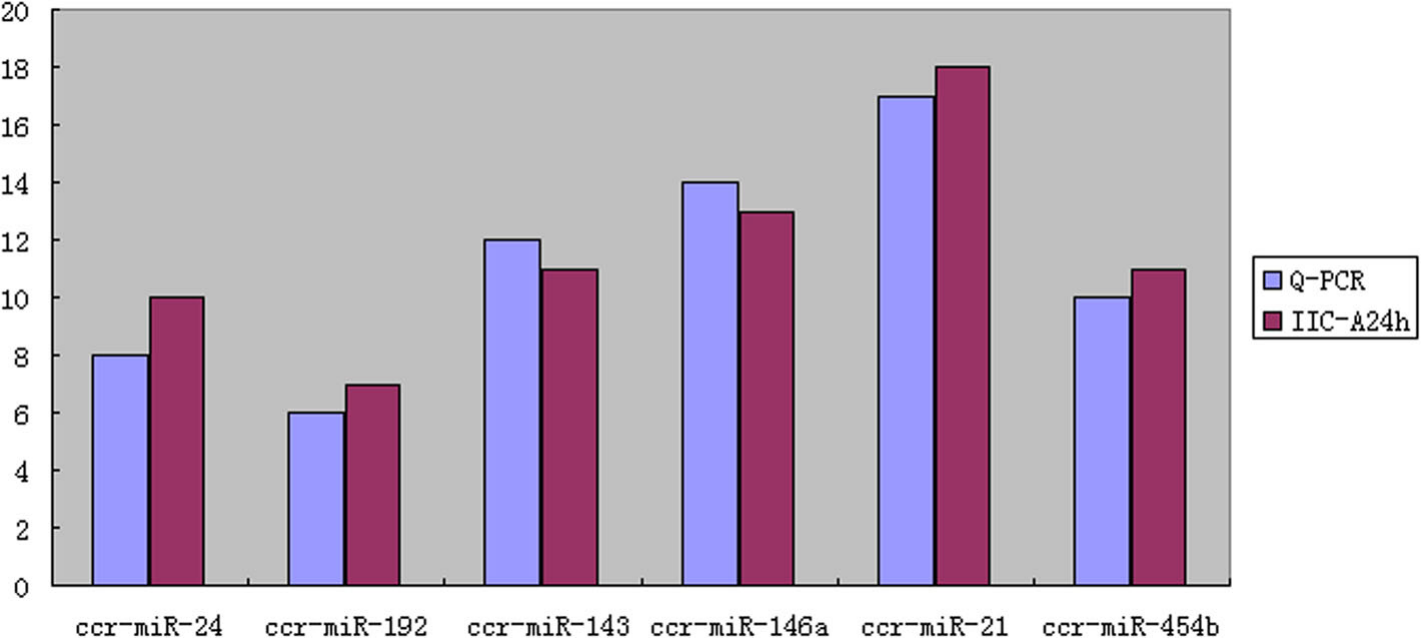
Real-time quantitative PCR gene expression analysis of six randomly-selected miRNAs. Gene expression was normalized to the level of U6 snRNA

To validate the results of Solexa sequencing, qPCR was used to test six randomly-selected (ccr-miR-24, ccr-miR-146a, ccr-miR-192, ccr-miR-21, ccr-miR-143, and ccr-miR-454b) miRNAs. According to sequence analysis, from the miRNAs selected for comparison, three miRNAs (ccr-miR-146a, ccr-miR-21, and ccr-miR-454b) were up-regulated in juvenile ovary gonad at 24 h whereas three miRNAs (ccr-miR-24, ccr-miR-192, and ccr-miR-143) were down-regulated in juvenile ovary at 24 h of atrazine exposure. The relative expression levels of all six miRNAs were consistent with the sequencing data (Fig. 3), indicating the reliability of the miRNA expression and correlation analysis.

### Effects of atrazine exposure on miRNA expression in PG of Yellow River carp

**Fig. 4:**
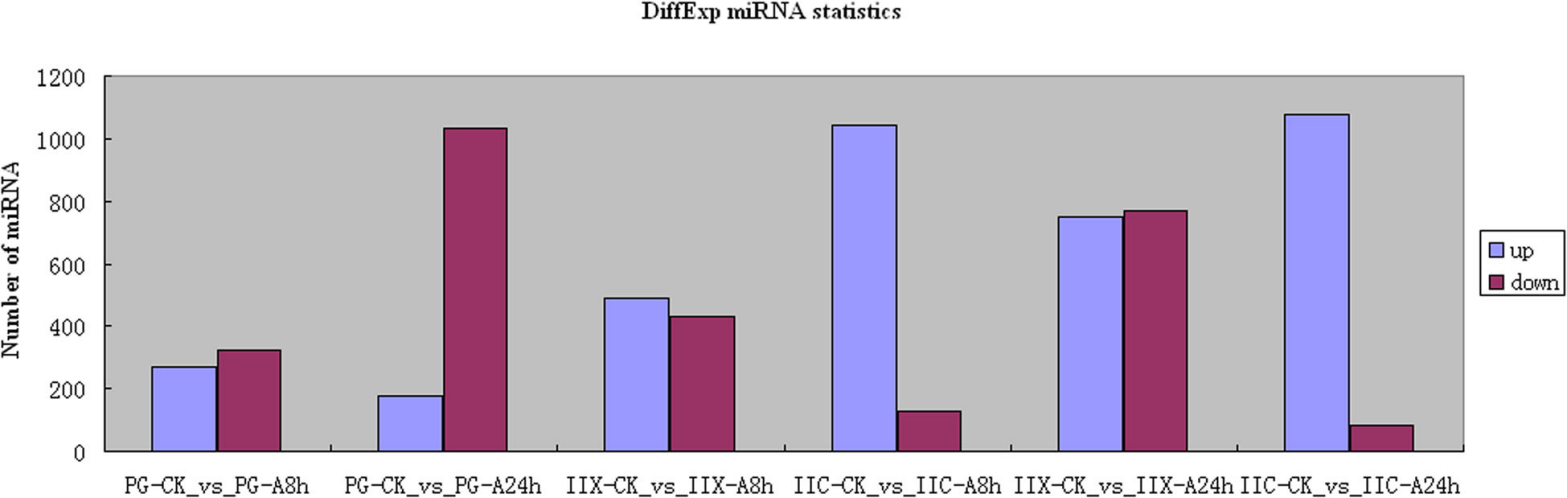
Differential expression of miRNAs in Yellow River carp. Greater than 2-fold change while *P* < 0.05

Primordial gonad is a critical period of sex differentiation, because of the formation of primordial germ cell. A comparative analysis of miRNA expression profiles with or without atrazine exposure may reveal miRNAs with important roles in early gonad differentiation. The results showed that atrazine exposure resulted in the altered expression of a larger number of miRNAs in PG compared with control. Atrazine exposure not only affected the total number of detectable miRNAs, but also the expression levels of miRNAs. After atrazine exposure for 8 h and 24 h, we observed different patterns of differentially-expressed miRNAs in PG of carp. Compared with the control group, 277 miRNAs were increased and 334 miRNAs were decreased after atrazine exposure for 8 h. A significant difference in miRNA expression was observed between samples from atrazine exposure for 24 h and unexposed controls, 181 miRNAs were increased and 1056 miRNAs were decreased (Fig. 4). The most significantly down-regulated miRNAs were miR-205, miR-184 and miR-203b-3p, which were down-regulated by 7.15, 3.61 and 3.35 fold, respectively. The most significantly up-regulated miRNAs were miR-7132, miR-135c, and miR-187 which were up-regulated by 8.70, 2.88 and 2.48 fold, respectively (Table 2). Atrazine exposure for 24 h had a greater effect on carp PG miRNA expression than the exposure for 8 h. The number of miRNAs with altered expression after atrazine exposure was higher at 24 h than at 8 h. However, the extent of change varied among the miRNAs. For example, the expression levels of miR-135c and miR-738 increased significantly (2.21- and 2.47-fold, respectively), whereas the expression levels of miR-203a decreased significantly (12.0-fold). Similarly, the changes in miRNA expression in PG varied between the unexposed control and atrazine exposure for 8 h or 24 h. For example, miR-135c was up-regulated by 2.2-fold after atrazine exposure for 8 h and was up-regulated by 2.8-fold after atrazine exposure for 24 h. MiR-122 was up-regulated by 1.4-fold after atrazine exposure for 8 h, but was down-regulated by 2.9-fold after atrazine exposure for 24 h. The miRNAs that were significantly altered in PG after exposure to atrazine may thus be involved in sex differentiation and development, and their importance in sex differentiation mechanisms needs to be clarified.

**Table 2:**
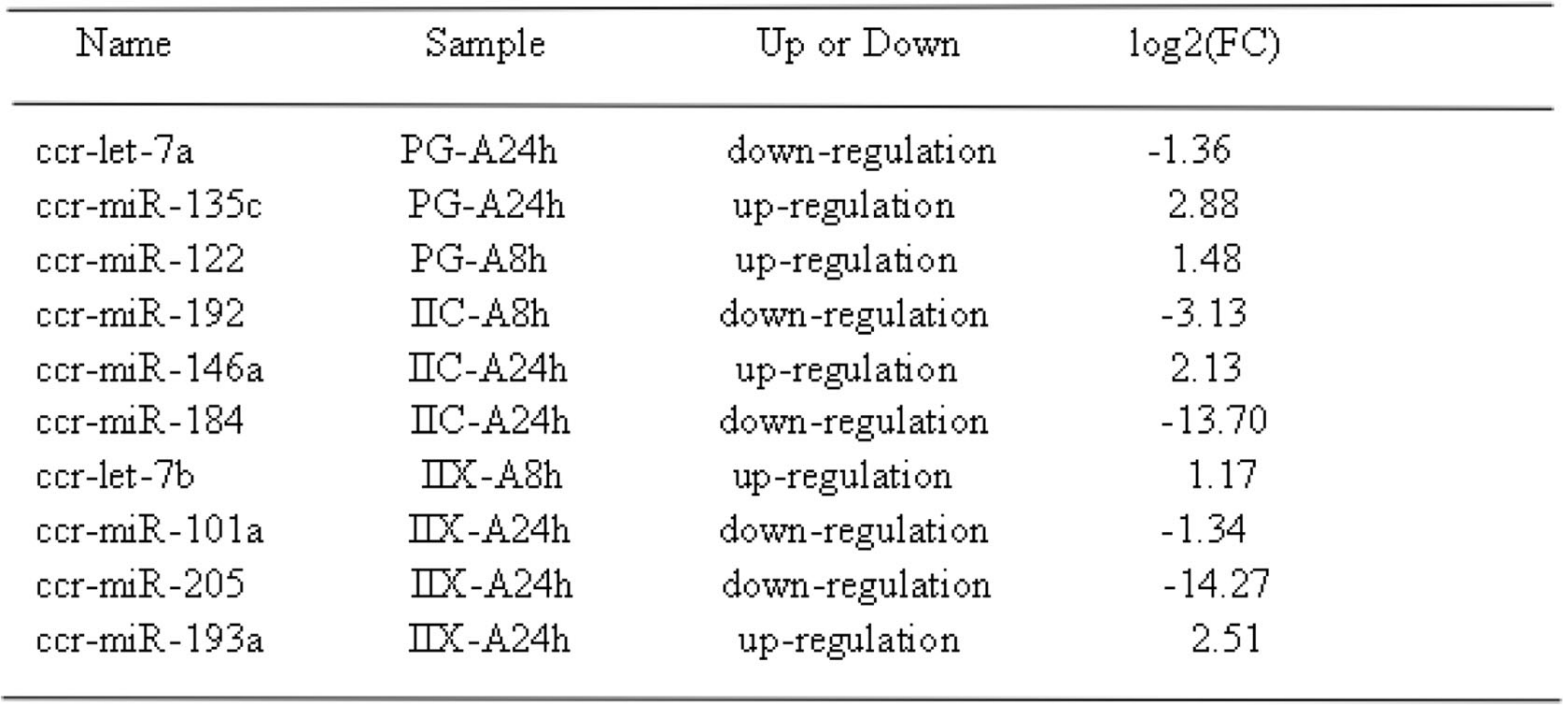
miRNAs with significant expression alterations after atrazine exposure in Yellow River Carp

### Effects of atrazine exposure on miRNA expression in juvenile gonad of Yellow River carp

We observed patterns of differentially-expressed miRNAs in juvenile gonad (stage II ovary and stage II testis) of carp after atrazine exposure for 8 h and 24 h, especially in juvenile ovary (Fig. 4). In juvenile ovary, 1053 miRNAs were increased and 132 miRNAs were decreased after atrazine exposure for 8 h, relative to unexposed controls. Relative to the control group, 1085 miRNAs were increased and 84 miRNAs were decreased after atrazine exposure for 24 h. The most significantly decreased miRNAs were miR-184, miR-214 and miR-122, which were decreased by 13.69, 13.21 and 12.40 fold respectively. The most significantly increased miRNAs were miR-17-3p, miR-454a, and miR-454b which were increased by 2.95, 2.49 and 2.42 fold respectively. In juvenile testis, 561 miRNAs were increased and 434 miRNAs were decreased after atrazine exposure for 8 h, relative to the control group. Compared with the control group, 775 miRNAs were increased and 799 miRNAs were decreased after atrazine exposure for 24 h. The most significantly down-regulated miRNAs were miR-205, miR-194, and miR-122, which were decreased by 14.27, 13.59, and 11.81 fold, respectively. The most significantly up-regulated miRNAs were miR-489, miR-738, and miR-193a, which were up-regulated by 10.61, 4.53, and 2.50 fold, respectively. Atrazine exposure for 24 h had a greater effect on juvenile testis miRNA expression than 8 h exposure. In addition, atrazine treatment led to a larger number of miRNAs with altered expression in juvenile testis, than in juvenile ovary. The number of down-regulated miRNAs was higher in juvenile testis than in ovary which is consistent with the feminizing effects of atrazine.

The extent of expression change varied among miRNAs. For example, in juvenile ovary, expression levels of miR-301a and miR-17-3p decreased by 1.38- and 2.95-fold after atrazine exposure for 24 h, respectively. In contrast, the miR-101b expression level decreased by 1.01-fold. In juvenile testis, expression levels of miR-193a and miR-146a increased by 2.50- and 1.71-fold, respectively, after atrazine exposure for with 24 h. In contrast, the expression levels of miR-122 decreased by 11.81-fold. Similarly, the changes in miRNA expression of juvenile ovary and testis varied between unexposed controls and atrazine exposure for 8 h or 24 h. For example, ccr-miR-210 was down-regulated by 1.36-fold after atrazine exposure for 8 h, and was down-regulated by 2.06-fold after atrazine exposure for 24 h in juvenile ovary. Ccr-miR-192 was down-regulated by 3.12-fold after atrazine exposure for 8 h, and was down-regulated by 4.07-fold after atrazine exposure for 24 h (Table 2). In juvenile testis, ccr-miR-205 was down-regulated by 3.50-fold after atrazine exposure for 8 h but was down-regulated by 14.27-fold after atrazine exposure for 24 h (Table 2). The miRNAs that were significantly altered in juvenile gonad after exposure to atrazine may thus be involved in sex differentiation and development, and their importance in sex differentiation mechanisms needs to be clarified.

### Expression patterns of miRNAs at different gonad developmental stages in Yellow River carp

**Fig. 5:**
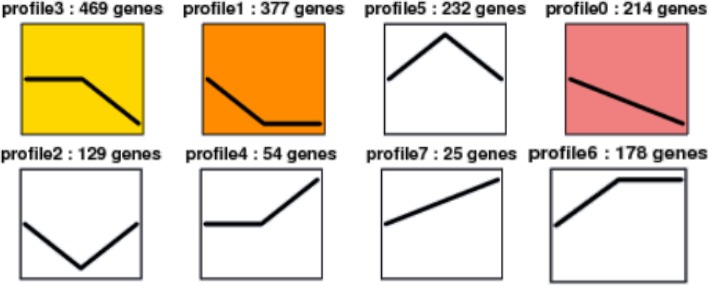
Trend analysis of miRNA expression profiles after exposure to atrazine in Yellow River Carp

Trend analysis of miRNA expression after exposure to atrazine for 8 h and 24 h, at different developmental stages, was conducted. In PG, we identified eight different expression patterns (Fig. 5), including 25 miRNAs that were up-regulated and 214 that were down-regulated during atrazine exposure (Fig. 5, profiles 3, 0). Expression of 232 miRNAs, such as miR-1 and miR-133a-3p, increased after exposure for 8 h, but decreased at 24 h (Fig. 5, profile 5). In contrast, 129 miRNAs, including miR-29a and miR-29b, showed the opposite expression pattern during atrazine exposure (Fig. 5, profile 2). In juvenile ovary, 8 different expression patterns (Fig. 5) were identified, including 440 miRNAs that were increased and 26 that were decreased during atrazine exposure (Fig. 5, profiles 7, 0). Expression of 157 miRNAs, such as mir-202-y and mir-27c-5p, increased after exposed for 8 h, but decreased at 24 h (Fig. 5, profile 5). In contrast, 70 miRNAs, including miR-155 and miR-92b, showed the opposite expression pattern during atrazine exposure (Fig. 5, profile 2). In juvenile testis, we also identified eight different expression patterns (Fig. 5), including 68 miRNAs that were up-regulated and 73 that were down-regulated during exposure (Fig. 5, profiles 7, 0). Expression of 117 miRNAs, such as mir-15a and mir-16a, increased after atrazine exposure for 8 h, but decreased at 24 h (Fig. 5, profile 5). In contrast, 41 miRNAs, including miR-144 and miR-148, showed the opposite expression pattern during atrazine exposure (Fig. 5, profile 2).

In this study, miRNAs targeting male-biased genes showed an upward trend. In PG, miR-499, which was predicted to target *sox9,* increased after exposure to atrazine (Fig. 5, profile 7). *Gsdf* which was targeted by miR-146a and of miR-22a, also increased after exposure to atrazine (Fig. 5 profile 7). The expression patterns of miR-72-x and miR-212-y, which were predicted to target *dmrt*, were also consistent with the above miRNAs which predicted male-biased target genes (Fig. 5, profile 7). In juvenile ovary, novel-m3245-5p, which was predicted to target *sox9*, increased after exposure to atrazine (Fig. 5, profile 7). *Gsdf* which was targeted by novel-m0192-3p and novel-m0514-3p, also increased after exposure to atrazine (Fig. 5, profile 7). MiR-454a and miR-454b, which were predicted to target *atm,* increased after exposure to atrazine (Fig. 5, profile 7). The expression patterns of novel-m0515-3p and novel-m0080-5p, which were predicted to target *dmrt*, were consistent with the above miRNAs which predicted male-biased target genes (Fig. 5, profile 18). In juvenile testis, novel-m3312-3p, which is predicted to target *sox9*, increased after exposure to atrazine (Fig. 5, profile 7). *Atm*, which was the predicted target of novel-m0167-3p and novel-m0417-3p, also increased after exposure to atrazine (Fig. 5, profile 7).

In contrast, miRNAs targeting female-biased genes showed a downward trend. Expression levels of novel-m0101-3p and novel-m3450-3p in PG, miR-101b in juvenile ovary, and miR-203b-3p in juvenile testis, all of which were predicted to target *Smad4*, decreased after exposure to atrazine (Fig. 5, profile 0). The most abundant differentially-expressed miRNAs after exposure to atrazine in PG, juvenile ovary and juvenile testis were let-7a, miR-143, and miR-125b, all of which decreased significantly during atrazine exposure.

The above results indicate that these miRNAs may influence gonad development.

### Identification and Signalling analysis of target genes of differentially-expressed miRNA

To identify potential targets of differentially-expressed miRNAs, involved in sex differentiation and development after atrazine exposure, we conducted target-gene prediction based on the common carp (*C. carpio*) genome sequence (http://www.carpbase.org/). A total of 26,299 genes were predicted to be the possible targets of 4353 differentially-expressed miRNAs that were commonly expressed in all atrazine exposure samples. Two hundred thirty-nine annotated signalling pathways were identified totally, including at least 10 pathways that involved in the reproductive process, for example *Wnt* signalling, Notch signalling, transforming growth factor-β (*TGF-β*) signalling, p53 signalling, steroid hormone biosynthesis, and estrogen signalling pathway. From the result, we found that the targets of 790 miRNAs belonged to the *MAPK* signalling pathway, which plays an important role in the process of spermatogenesis in the testis. In addition, *MAPK* signalling has been reported to be involved in the acrosome reaction in the female reproductive tract before fertilization of the ovum [55]. *Wnt* signalling was reported to be involved in mammalian reproduction [56], and in zebrafish sex determination [57]. We detected 415 miRNA targets belonging to the *Wnt* signalling pathway, 30 belonging to *NF-kappa B* signalling pathway, and 133 belonging to p53 signalling pathway. *Wnt* signalling pathway, *NF-kappa B* signalling pathway and p53 signalling pathways were associated with sex differentiation in zebrafish [57]. Target genes predicted to belong to the three pathways in our study may be involved in sex differentiation and gonad development in Yellow River carp. Moreover, we identified 245 miRNA targets belonging to the *TGF-β* signalling pathway, and 179 belonging to the Notch signalling pathway. In addition, we also identified 31 miRNA targets belonging to oestrogen signalling pathway, which may play an important role in hormone regulation.

To determine the key biological process of the putative target genes related to atrazine exposure, GO analysis was performed. The identified biological processes that the putative target genes were classified into include reproduction, reproductive process, response to stimulus, developmental process, and growth, which were all mechanisms related to sex differentiation and gonad development. The results showed possible relationships between atrazine, putative targets and gonad development, and suggested that atrazine may have effect on sex differentiation and gonad development.

We analysed the relationships between differentially-expressed miRNAs and their putative target genes. *Foxl2*, *gsdf*, *sf1*, *dmrt* and *stat1* have been shown to be essential factors in early ovary differentiation [58, 59]. *Smad4*, *atm*, *sox9*, and *smad3*, which are also known to be responsible for gonad differentiation, were also analysed. We found that these genes were predicted to be the target genes of multiple miRNAs, and thus negatively regulated by these targets. Given the important roles of steroid hormones in sexual dimorphism and reproduction in fish, we analysed the relationships between miRNAs, including *hsd11b* and *hsd3b*, mRNAs, and the steroid hormone biosynthesis pathway. Atrazine has endocrine-disrupting effects by altering the HPG axis [60]. We analysed genes that have critical roles in the regulation of the HPG axis including *ER1*, *ER2*, *AR*, and *CYP19A1*.

In the PG, a higher number of miRNAs targeting female-biased genes were down-regulated. MiR-135c which was significantly down-regulated by 2.21-fold and 2.88-fold after exposure to atrazine for 8 h and 24 h, respectively, were predicted to target *ER*, *foxl2*, and *CYP19A*. *Gsdf* was predicted to be the target of miR-132a, miR-146a, miR-210, and miR-22a which were also down-regulated. Our results indicated that atrazine can promote early gonad-determining genes by down-regulating miRNAs. MiR-205, which was predicted to target *atm*, *EGF*, *bcl2*, *BMP1* (bone morphogenetic protein 1), was significantly up-regulated by 7.23-fold for 8 h and 7.15-fold for 24 h, respectively. MiR-132a, which targeted *dmrt2*, was up-regulated by 1.22-fold after exposure to atrazine for 24 h, but it was not at 8 h. miR-499, which also targeted *dmrt2*, was up-regulated by 1.46-fold for 8 h and 1.57-fold for 24 h, respectively. After exposure to atrazine for 24 h, miR-202x, and miR-374-y, which were predicted to target *smad3*, were up-regulated and down-regulated, respectively. *Hsd11b* was predicted to be the target of miR-216-x and miR-342-y. *Hsd3b* was the predicted target of let-7-z. *Stat1* was the predicted target of miR-135c and miR-430, *Sf1* of miR-154-y and miR-3958-y, and *Sox9* of miR-499. The above results indicate the possible roles of the differentially-expressed miRNAs in PG, after exposure to atrazine during gonad differentiation.

In juvenile ovary, miR-21, which was significantly up-regulated by 2.18-fold after exposure to atrazine for 24 h, was predicted to target *AR* and *atm*. MiR-101b, which was predicted to target *sf1*, was significantly down-regulated by 1.01-fold. MiR-132a, which was significantly up-regulated by 1.08-fold after exposure to atrazine for 8 h, was predicted to target *AR*, *dmrt2*, *gsdf*, and *atm*. *Smad4* was predicted to target novel-m0048-5p. *Hsd11b* was predicted to target novel-m0305-3p. *Hsd3b* was predicted to be the target of miR-410-x. *Stat1* was the predicted target of miR-192, *CYP19A* of miR-203a and novel-m0527-3p, and *Sox9* of novel-m0011-5p.

In juvenile testis, miR-181b, and miR-181c, which were significantly up-regulated by 1.04-fold and 1.41-fold, respectively, after exposure to atrazine for 24 h, was predicted to target *dmrt2* and *atm*. miR-146a, which was predicted to target *gsdf* was significantly up-regulated by 1.70-fold. MiR-132a, which was significantly down-regulated by 1.28-fold, was predicted to target *ER*. *Smad4* was predicted to target miR-200b. *Hsd11b* was predicted to target novel-m0305-3p. *Hsd3b* was predicted to be the target of miR-410-x. *Stat1* was the predicted target of miR-192, *CYP19A* of miR-203b-3p and novel-m0693-5p, and *Sox9* of novel-m0081-5p. These results indicate that atrazine promotes the biosynthesis of steroid hormone by altering the miRNAs.

These differentially-expressed miRNAs were also predicted to play key roles in many reproductive biology pathways, including *TGF-β* receptor signalling, *Wnt* signalling, steroid metabolic processes, and cell differentiation. Moreover after exposure to atrazine for 24 h, the predicted target genes of the differentially-expressed miRNAs of PG included *hsd3*, *lemd3*, *grk6*, *ccna1*, *pcna*, *smad4*, *tbx6*, *GATA*, *cyp51a1*,*RBMS1*, *zranb1*, and *prosapip1*, and some of which, such as *hsd3* and *cyp51a1*, are gonad development-related genes. Besides, many other miRNAs were predicted to target genes involved in reproductive processes. MiR-205 and miR-135c were predicted to be the target of *bcl2* and *notch2*, which belong to the *TGF-β* signalling and Notch signalling pathways, respectively. MiR-205 was predicted to target *inhibin beta A chain* and *pdk1*, which are related to female gonad development, and *TGF-β* signalling, respectively. The results indicate that the differentially-expressed miRNAs perform some of the possible roles of in gonad reproductive processes. However, the specific mechanism needs further experimental verification.

## Discussion

MiRNAs are involved in diverse biogenesis pathways and have versatile regulatory functions in differentiation, proliferation, and apoptosis [61]. To date only a limited number of studies have investigated miRNA expression alterations in response to exposure to endocrine-disrupting chemical in fish and humans [62–65]. There are only few reports on the miRNA profiling of fish, in response to atrazine exposure, mainly focus on zebrafish and no reports in common carp [17, 18, 21, 22, 26, 66, 67]. The investigation into the adverse effects of atrazine exposure on miRNAs is important to reveal the molecular mechanism of gonad differentiation. In the present study, we assessed the potential effects of atrazine on miRNAs in the reproductive system at two developmental stages (PG and II-stage gonad) of Yellow River carp. Primordial germ cell formation is a crucial stage of gonad differentiation, and II-stage gonad is the stage of evident sex differentiation. Comparative analysis of miRNA expression profiles at these two important stages, after exposure to atrazine, is helpful to identify miRNAs that play important roles in gonad differentiation.

In this study, atrazine exposure resulted in changes in the sex ratio of carp, the ratio of female increased with the increase of concentration. In addition, atrazine exposure resulted in significant expression alterations of various miRNAs. Atrazine exposure for 24 h caused more alterations in the expression of miRNAs than exposure for 8 h. Atrazine exposure for 24 h caused more alteration in miRNA expression in juvenile testis than in juvenile ovary. It is thus clear that acute and short-time exposure to atrazine during development can produce adverse effects, as has been suggested before [68].

Several studies in amphibians have suggested that atrazine is associated with feminization of males in the wild [20, 69]. In field studies, atrazine has repeatedly been associated with the presence of feminized secondary sex characteristics in male frogs [70]. In fish, atrazine causes degeneration of interstitial tissue in the testes [66] and feminizes the gonads of developing male teleost fish [71]. In addition, embryonic atrazine exposure alters the expression of zebrafish and human miRNAs known to play a role in angiogenesis, cancer, neuronal development, differentiation, and maturation [22]. In our study, atrazine exposure altered the expression of carp miRNAs that play a role in gonad differentiation and gonad development. A number of miRNAs, such as let-7, miR-21, miR-101, and miR-124 that are highly expressed in silver carp and adult bighead carp were significantly altered in our study [72].

Our results suggest that miR-181a, miR-21, miR-430, let-7, and miR-143 may involve in the process of gonad differentiation and development in carp.

Several studies have shown that miR-21 play an important role in gonad differentiation and development. A study reported that in cattle miR-21 was significantly up-regulated in the ovary (relative to testis), which suggest that miR-21 may play a regulatory role in female physiology [73]. Other study showed that miR-21 also plays a certain role in preventing periovulatory granulosa cells apoptosis when they transit into luteal cells [74]. In addition, has-miR-21 was also shown to be increased by ovarian steroids in mouse granulosa cells, glandular epithelial cells, and human endometrial stromal cells [75, 76]. In the present study, atrazine exposure did not change the expression of miR-21 in the PG after atrazine exposure, but induced its up-regulation in juvenile ovary and down-regulation in juvenile testis.

The predicted targets of miR-21 included genes in the *TGF-β*, B-cell receptor, *MAPK*, and apoptotic pathways. This observation suggests that miR-21 may play crucial roles in ovary development, gonad differentiation [77], and endocrine regulation [55, 78]. The predicted target genes of miR-21 in our study were *AR* and *atm*.

Let-7 was another family of miRNAs with altered expression by atrazine exposure. The let-7 family was first discovered and characterized in *Caenorhabditis elegans*, and plays an important role in regulating late developmental events by down-regulating lin-41, and possibly other genes [79]. Let-7 was significantly up-regulated after atrazine exposure in the PG and juvenile testis. The predicted target genes of let-7 in our study were *sox9* and *atm*.

The miR-430 family was reported to have important role in embryonic morphogenesis and clearance of maternal mRNAs, and it’s highly expressed during early zebrafish development [67, 80–82]. Study has shown that miR-430 target chemokine signalling to ensure accurate migration of primordial germ cells [83]. In our study and miR-430 was down-regulated in PG but not in juvenile gonad, which indicates that miR-430 has an important role in early gonad differentiation of Yellow River carp.

Several reports showed that miR-143 is highly expressed in the juvenile ovary; it is a dominant miRNA in ovaries in yellow catfish, pigs, and cattle [84, 85]. In this study, miR-143 was highly expressed in the ovary of common carp exposed to atrazine, which is in keeping with previous reports.

The miR-181a family is abundantly expressed in the gonads of tilapia [86], mice [87], and humans [88]. It was down-regulated in juvenile ovary in the present study. Overall, above results suggest that miR-21, let-7, miR-430, miR-181a, and miR-143 may play vital roles in gonad development process in Yellow River carp.

Differentially expressed miRNAs showed a variety of expression patterns at different development stages. Among the 8 different expression patterns, two patterns are worthy of attention, involving miRNAs with expression levels that either up-regulated or down-regulated significantly after atrazine exposure. MiRNAs whose expression either increased or decreased significantly after atrazine exposure may be direct regulators of gonad differentiation. Samples with the highest number of miRNAs with altered expression were the PG and juvenile ovary exposed to atrazine for 8 h or 24 h. The number of decreased miRNAs was 1056 in PG, including miRNAs which targets were female-biased. Because miRNAs are negatively correlated with its target genes, this observation suggests that atrazine promotes the expression of female-biased genes by decreasing specific miRNAs in PG, which would result in the differentiation of the gonad to the female phenotype. The juvenile ovaries exposed to atrazine had the highest number of up-regulated miRNAs, including miRNAs whose targets are male-biased. It is thus possible that atrazine represses the expression of male-biased genes by increasing specific miRNAs in juvenile ovary.

The juvenile testis exposed to atrazine had the highest number of miRNAs with altered expression, indicating that this tissue was more sensitive to atrazine, possibly leading to the feminization of males. This observation suggests that these miRNAs may have an important function in the timing of gonad differentiation and development.

The result of target prediction showed that many of the miRNA targets that we identified were involved in gonad differentiation.

Among these predicted target genes, *dmrt*, *gsdf*, and *sox9* have been identified as sex-determining genes in fish [89, 90]. For example, *Hsd11b* and *hsd3b* which encoding key enzymes in the steroid hormone biosynthesis pathway, may involved in steroid hormone synthesis, sex differentiation, and gonad function, and may play a vital role in developmental timing. However, further studies are needed to confirm the interactions and functions of miRNA and target genes. In addition, the results also show that atrazine has oestrogenic effects down-regulating male-biased genes (such as *dmrt* and *atm*) through specific miRNAs up-regulation, and up-regulating female-biased genes (such as *foxl2*) through specific miRNAs down-regulation.

Previous studies showed that atrazine exposure can significantly reduce synthesis, secretion, and the circulating levels of androgens in fish [66, 91], amphibians [20, 11], reptiles [92], and mammals [93, 94], and also in birds to a lower extent [95]. The endocrine-disrupting effects of atrazine are primarily due to alterations of the HPG axis [17, 25, 26, 24, 23]. However, atrazine’s mechanism of action is not well-understood, it has been proposed that atrazine up-regulate aromatase expression [96–99]. Aromatase up-regulation leads to increased conversion of androgens into oestrogens [100]. In the present study, we analysed genes that regulate hormone biosynthesis in the HPG axis, including *ER1*, *ER2*, *AR*, and *CYP19A1*. MiR-122, which targets *ER1* and *ER2*, was down-regulated by atrazine. MiR-21, which targets *AR* was up-regulated in PG by atrazine. MiR-203a, which targets *CYP19A1,* was down-regulated in PG by atrazine. Our results indicate that atrazine possibly can up-regulate aromatase expression through specific miRNAs, which is consistent with previous studies.

We tested the hypothesis that atrazine has endocrine-disrupting effects by altering genes of the HPG axis through its corresponding miRNAs. In the PG, atrazine affects sex differentiation mainly through altering upstream genes involved in gonad differentiation. In juvenile ovary or testis, atrazine affects the gonad development mainly through altering hormone generation and the expression of hormone receptor genes. Further studies are needed to investigate the mechanisms and roles of miRNAs in the regulation of genes during gonad differentiation and development.

## Conclusions

Atrazine is widely used in agriculture and is a known endocrine disrupting chemical, but the effects of atrazine on the reproduction of carp, especially miRNA have not been investigated. In this study, the effects of atrazine on the reproduction of carp, especially miRNA were analyzed. Atrazine exposure can affect the sex ratio, and caused significant alterations in miRNAs expression at the crucial stages of carp gonad development. Target genes of differentially-expressed miRNAs are key factors in early ovary differentiation or play an important role in the formation of germ cells. Our results indicate that atrazine promoted the expression of female-biased genes by decreasing miRNAs in primordial gonad. In addition, our results indicate that atrazine can up-regulate aromatase expression through miRNAs, which supports the hypothesis that atrazine has endocrine-disrupting activity by altering the expression of genes of the Hypothalamus-Pituitary-Gonad axis through its corresponding miRNAs.

## Methods

### Chemicals

Atrazine (purity > 98%) was purchased from Beijing Dezhong-Venture Pharmaceutical Technology Development Co., Ltd. (Beijing, China). As atrazine has low solubility in water, the stock solutions and dilutions were prepared in acetone (Fisher Scientific, USA) and stored at 4 °C.

### Fish samples

The sample fish (Yellow River carp) used in this study were obtained from the aquaculture facilities of Henan Normal University and maintained at the genetics laboratory (Henan normal university, Xinxiang Henan province, China) in flow-through water tanks with a constant temperature of 25 ± 1 °C. Embryos were obtained by natural spawning and larvae were cultured in embryo medium following standard procedures. Fry were fed two times daily with commercial flake food. All investigations in this study were performed according to the Animal Experimental Guidelines of the Ethical Committee of the University of China.

The experimental sample of miRNA expression profile included gonads from two different developmental stages. According to the results of our previous studies, primordial gonad samples were collected from larvae at 45 days post-hatching [49]. The original reproductive gonad was dissected under a microscope, and samples from 50 fish were mixed after confirmation by histological section. Samples of juvenile gonad were collected from 30 fish 80 days post-hatching. Stage II ovaries and testis were confirmed with histological sections.

### Atrazine exposure

At 5 days post-hatch (dph), fry were divided into several groups, held in the water containing atrazine at a concentration of 0, 4.28, 42.8, or 428 μg/L. To avoid metabolic and microbial breakdown of atrazine, half of the water was removed and replaced every 3 days with fresh atrazine-contaminated water. At 130 dph, 100 fish from each group were randomly selected for histological examination of the sex.

Samples of two different stages including primordial gonad and juvenile gonad (ovary and testis) were cultured at 28 °C in a humidified 10% CO_2_ atmosphere in Dulbecco’s modified eagle medium supplemented with 10% foetal bovine serum (Gibco, Life Technologies) [101, 102]. Culture medium was renewed every 2 days. Before gonad collection the fish were anesthetized by immersion in 50 μg/ml of tricaine methane sulfonate (MS-222). And at the end of the experiment fish were euthanized by immersion into a 0.5 g/L tricaine solution (Sigma-Aldrich). For atrazine exposure experiments, cells were seeded in 24-well plates and allowed to proliferate for 48 h. Then samples were treated with 428 μg/L of atrazine for 24 h. Three replicates were set for each treatment, as well as for the unexposed control. Samples were collected at 8 h and 24 h post-treatment, and were immediately frozen in liquid nitrogen for further use.

### RNA isolation

Total RNA was extracted from each sample separately using TRIzol reagent (Invitrogen, Carlsbad, CA, USA) according to the instructions. The quantity and purity of total RNA were checked using the Agilent 2100 Bioanalyzer system (Santa Clara, CA, USA) and by denaturing gel electrophoresis. The total RNAs were then stored at − 80 °C for further use.

### Small-RNA library construction and sequencing

Small-RNA libraries were generated from the nine samples of Yellow River carp: primordial gonad control (PG-CK), primordial gonad exposed to atrazine for 8 h (PG-A8h), primordial gonad exposed to atrazine for 24 h (PG-A24h), juvenile ovary control (IIC-CK), juvenile ovary exposed to atrazine for 8 h (IIC-A8h), juvenile ovary exposed to atrazine for 24 h (IIC-A24h), juvenile testis control (IIX-CK), juvenile testis exposed to atrazine for 8 h (IIX-A8h), juvenile testis exposed to atrazine for 24 h (IIX-A24h). Small-RNA libraries were generated using the mirVanaTM mircoRNA Isolation Kit (Ambion, USA), according to the manufacturer’s instructions. Small-RNA libraries were prepared from three biological replicates for each sample.

Total RNA was ligated with 3′ and 5′ RNA adaptors. Fragments with adaptors on both ends were enriched by PCR after reverse transcription, as described previously [49]. The resulting cDNAs were purified and enriched with 6% denaturing polyacrylamide gel electrophoresis to isolate the fractions of the expected size and to eliminate unincorporated primers, primer dimer products, and dimerized adaptors [49]. Finally, the nine resulting RNA libraries were sequenced using an Illumina/Solexa Genome Analyzer, at Guangzhou Genedenovo Biotech Company (Guangzhou, China).

### Sequencing data analysis

As we described previously [49], after remove low quality reads, adaptor sequences and adaptor trimming, reads of 16–35 nt in length were kept for further bioinformatic analysis. Using Bowtie (version 1.1.0), the remaining reads were mapped to the *C. carpio* genome with a tolerance of zero mismatches in the seed sequence. By blasting against the Rfam (11.0, http://rfam.xfam.org) and GenBank (http://www.blast.nvbi.nlm.nih.gov/) databases, the reads mapped to the *C. carpio* genome were then analysed to annotate snRNA, tRNA, rRNA, snoRNA, and non-coding RNA sequences. The remaining sequences were identified as the conserved miRNAs in carp by blasting against miRBase 21.0 allowing no more than two mismatches. Existing carp miRNAs referring to *C. carpio* miRNA were included in the miRBase with no base mismatch. The sequences that did not match existing or conserved miRNAs were used to identify potentially novel miRNA candidates [103, 104]. Novel miRNA candidates were identified by folding the flanking genome sequence of unique small RNAs using MIREAP (https://sourceforge.net/projects/mireap/). The enrichment level of each miRNA was identified by counting the number of reads in each sample. To identify differentially-expressed miRNAs within the nine libraries, the frequency of miRNA counts was normalized as transcripts per million (TPM). The TPM values were calculated as follows: normalized expression, TPM = (actual miRNA count/number of total clean reads) × 1,000,000. Only the miRNAs with over 2-fold changes in the two compared samples were considered differentially-expressed miRNAs (*P* < 0.05) [105]. A positive value represents up-regulation of a miRNA, while a negative value indicates down-regulation.

### Prediction of miRNA targets

Targets of miRNAs were predicted using three softwares, including miRanda (v3.3a), RNAhybrid (v2.1.2) + svm light (v6.01) and Targetscan. The overlap of the predicted results was considered to represent the final result of predicted target mRNAs.

### Gene ontology (GO) and pathway analysis of atrazine-responsive mRNA targets

Pathway analysis of the predicted target mRNAs was performed using the Kyoto Encyclopedia of Genes and Genomes (KEGG) pathway database (http://www.genome.jp/kegg/pathway.html) [106]. To classify the selected genes into groups with similar patterns of expression, each gene was assigned to an appropriate category, according to its main cellular function. To determine the biological phenomena target mRNAs were involved in, the DAVID (http://david.abcc.ncifcrf.gov/home.jsp) functional annotation clustering tool was used.

### qPCR for validation of miRNAs

The expression profiles of six randomly-selected miRNAs were investigated with qPCR to validate their expression changes. Total RNA (500 ng) was converted to cDNA using miScript reverse transcriptase mix (Qiagen, Valencia, CA, USA) according to the manufacturer’s instructions. qPCR was carried out using an Applied Biosystems 7300 Real-Time PCR System according to the standard protocol. CDNA samples were diluted to 1:150; 5 μL were used for each real-time PCR reaction. The 20-μL PCR mixture included 10 μL SYBR Premix Taq (2×), 0.4 μL miRNA-specific forward primers (10 μM), 0.4 μL miScript universal primer (10 μM), and 1 μL PCR template (cDNA). The PCR thermal program was 50 °C for 2 min, followed by 40 cycles of 95 °C for 2 min, 95 °C for 15 s, and 60 °C for 30 s. Melting curve analysis was performed after amplification. Standard curves for endogenous control and for all miRNAs were constructed using serial dilutions of a pooled cDNA sample. Standard curves were used to determine the quantity of the selected miRNAs and reference genes. Relative miRNA expression levels were calculated using the 2^−ΔΔCt^ method. Each sample was run in triplicate. SnRNA *U6* was used as an endogenous control for qPCR of miRNAs.

## Additional files

Additional file 1: Differentially expressed miRNAs after exposed to atrazine. (XLS 510 kb)

Additional file 2: Predicted target genes of differentially expressed miRNAs and target genes associated with significantly enriched GO terms after exposed to atrazine. (XLS 80605 kb)

Additional file 3: Differentially expressed mRNAs of PGC stage after exposed to atrazine for 8 h. (XLS 14039 kb)

Additional file 4: Differentially expressed mRNAs of PGC stage after exposed to atrazine for 24 h. (XLS 13978 kb)

Additional file 5: Differentially expressed mRNAs of juvenile ovary after exposed to atrazine for 8 h. (XLS 20365 kb)

Additional file 6: Differentially expressed mRNAs of juvenile ovary after exposed to atrazine for 24 h. (XLS 20271 kb)

Additional file 7: Differentially expressed mRNAs of juvenile testis after exposed to atrazine for 8 h. (XLS 24312 kb)

Additional file 8: Differentially expressed mRNAs of juvenile testis after exposed to atrazine for 24 h. (XLS 24795 kb)

## Data Availability

The deep sequencing data sets generated in this project and supporting the results of this article are included within the article and its Additional files 1, 2, 3, 4, 5, 6, 7 and 8.
